# Predominance of *L. monocytogenes* Lineage I Clones in Wastewater, Ruminants, and Natural Environments

**DOI:** 10.1111/1462-2920.70169

**Published:** 2025-09-07

**Authors:** Yuval Markovich, Alexandra Moura, Jesús Gomis, Alexandre Leclercq, Ángel Gómez‐Martín, Hélène Bracq‐Dieye, Carla Palacios‐Gorba, Nathalie Tessaud‐Rita, Susana Ortolá, Guillaume Vales, M‐Adela Yáñez, Pierre Thouvenot, Philippe Pérot, Marc Lecuit, Juan J. Quereda

**Affiliations:** ^1^ *Listeria*: Biology and Infection Research Group (LisBio) Valencia Spain; ^2^ Departamento Producción y Sanidad Animal, Salud Pública Veterinaria y Ciencia y Tecnología de los Alimentos Facultad de Veterinaria, Universidad Cardenal Herrera‐CEU, CEU Universities Valencia Spain; ^3^ Institut Pasteur, *Listeria* National Reference Centre and WHO Collaborating Centre Paris France; ^4^ Institut Pasteur, Université Paris Cité, Inserm U1117, Biology of Infection Unit Paris France; ^5^ Microbiological Agents Associated With Animal Reproduction (ProVaginBIO) Research Group Valencia Spain; ^6^ Public Health Laboratory of Valencia Valencia Spain; ^7^ LABAQUA SAU Alicante Spain; ^8^ Division of Infectious Diseases and Tropical Medicine, Institut Imagine, APHP Necker‐Enfants Malades University Hospital Paris France

**Keywords:** cgMLST, ecology, foodborne, pathogens, persistence, virulence, whole genome sequencing

## Abstract

*Listeria monocytogenes* is a saprophytic bacterium and a foodborne pathogen of humans and animals. Little is known about its distribution and genetic diversity across different environments within the same geographical region. We conducted a large‐scale longitudinal study in southeastern Spain monitoring *Listeria* spp. in untreated wastewater, ruminant farms, and natural environments over four seasons (*N* = 1490 samples, *N* = 545 isolates) and in food and food‐processing environments (*N* = 7395 samples, *N* = 255 isolates). *Listeria* spp. were more abundant in host‐associated than natural environments, and non‐pathogenic *Listeria* were more prevalent than *L. monocytogenes* in both niches. *L. monocytogenes* was detected in 42.7%, 11.4%, 4.2%, and 3.4% of wastewater, ruminant farms, natural environments, and food‐related samples, respectively. Hypervirulent lineage I accounted for 82.9% of *L. monocytogenes* isolates from wastewater, ruminant farms, and natural environments, while lineage II represented 74.1% in food‐related samples. Among 255 *L. monocytogenes* cgMLST types, 5% were shared across environments, demonstrating circulation between different environments. Persistent *L. monocytogenes* clones were detected in food processing environments and ruminant farms. Our data suggest anthropogenic activities and livestock drive *Listeria* spp. dissemination. These results provide insights into the interactions of *Listeria* spp. in the environment, improving surveillance strategies to reduce pathogen transmission, food contamination, and clinical cases.

## Graphical abstract

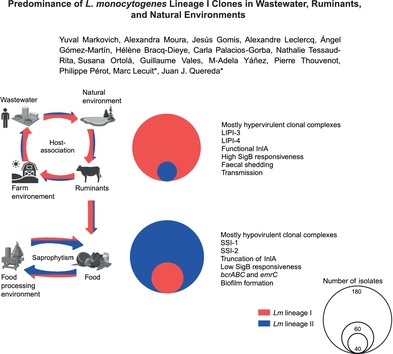

## 1 Introduction

The genus *Listeria* comprises 28 species of widely distributed, small rod‐shaped Gram‐positive bacteria (Quereda et al. 2021; Raufu et al. 2022). Only *Listeria monocytogenes* and *Listeria ivanovii* are zoonotic pathogens (Orsi and Wiedmann 2016). *L. monocytogenes* is an opportunistic entero‐invasive gastrointestinal pathogen. It can cause listeriosis, a potentially fatal foodborne infection in humans with a high case fatality rate (18.1%) (Charlier et al. 2017; EFSA 2023) that also affects other animal species, including ruminants (Quereda et al. 2021). In immunocompetent individuals, listeriosis results in a self‐limited febrile gastroenteritis (Quereda et al. 2021). In the elderly and immunocompromised individuals, listeriosis can manifest as septicemia and meningoencephalitis, while pregnancy is at particular risk for foetoplacental infection, preterm birth, abortion, and neonatal infection (Charlier et al. 2017). In animals, listeriosis most commonly manifests as septicemia and rhombencephalitis and can cause stillbirth and abortion (Quereda et al. 2021).

*L. monocytogenes* is detected in 10% of healthy human stool samples (Hafner et al. 2021). Human exposure to *L. monocytogenes* is mainly attributed to the consumption of contaminated food (Quereda et al. 2021; Disson et al. 2021). Livestock animals can become infected through ingestion of contaminated feed and contact with contaminated farm environments such as bedding, food, and water troughs (Palacios‐Gorba et al. 2021; Wiedmann et al. 1996). Wastewater monitoring for pathogens is an effective tool because it captures them as they are excreted in faeces or urine (Paillard et al. 2005; Diamond et al. 2022). Wastewater may play an important role in the current ecology of *Listeria* because it can resist conventional wastewater treatment processes (Paillard et al. 2005). Discharge of contaminated wastewater and animal manure into natural and agricultural environments is likely to be associated with the habitat enrichment of *Listeria* spp. in soil and vegetation (Paillard et al. 2005; Lourenco et al. 2022; Rodriguez et al. 2021). *L. monocytogenes* is highly adapted to a wide range of stress conditions. It has been suggested that the presence of disinfectant resistance genes in *L. monocytogenes* increases its presence in food processing environments (NicAogáin and O'Byrne 2016).

Lineage I *L. monocytogenes* isolates, particularly those of clonal complex (CC) CC1, CC2, CC4, and CC6, have been reported to be hypervirulent and associated with human clinical cases and outbreaks, whereas lineage II isolates tend to be hypovirulent, particularly those belonging to CC9 and CC121, and associated with food and food processing environments (Hafner et al. 2024; Maury et al. 2016; Orsi et al. 2011). *L. monocytogenes* alternates between a saprophytic and a host‐associated lifestyle (Quereda et al. 2021). Some virulence factors, such as LIPI‐3 and LIPI‐4, are carried by hypervirulent clones that better colonise the intestinal lumen and invade more intestinal tissues than hypovirulent clones, reflecting their adaptation to the host (Hafner et al. 2024; Maury et al. 2016, 2019). Systematic characterisation studies of *L. monocytogenes* clones circulating among humans, livestock, the natural environment, and the food and food processing environment are important to elucidate the ecology of the pathogen and understand its circulation between these niches.

The objectives of the present study were (i) to determine the prevalence of *Listeria* spp. in urban wastewater, ruminant farms, and natural environments by a 1‐year longitudinal study design (2021–2022) in the same region, thus determining the ecological preferences of the genus; (ii) to compare the prevalence of *L. monocytogenes* in these environments with the prevalence of *L. monocytogenes* in food and food processing environments of the same geographic region over a longer period of time (2019–2023); (iii) to characterise the genetic diversity and population structure of all *Listeria* spp. isolates using whole‐genome sequencing (WGS); (iv) to determine whether *L. monocytogenes* clones circulate and persist among all sampled environments; and (v) to determine whether there is a seasonal variation in *L. monocytogenes* prevalence in the environmental niches studied.

## 2 Materials and Methods

### 2.1 Sample Collection

Samples were collected over 1 year during four consecutive seasons (March 2021 to March 2022) from multiple locations in southeast Spain. Each sampling area was visited four times, once per season. Wastewater sampling was conducted twice per month over 1 year.

- **Wastewater treatment plants**. In collaboration with 4 treatment plants, untreated influents from urban environments were analysed (Table S1). A total of 234 samples were analysed. The samples were transported to the laboratory under cooling conditions and were analysed immediately upon arrival.
- **Ruminant farms**. Three dairy ruminant farms (bovine, ovine, and caprine) and one mixed teaching farm (bovine and ovine) were sampled (Table S1). Clinical listeriosis was not detected in either of the farms before and during the sampling period. On each farm visit, 50 samples were collected (32 faeces samples of random individual animals and 18 samples from the farm environment, including feed (X3), bedding (X3), water trough (X3), food trough (X3), farm machinery (X3), and milking station (X3) from the dairy bovine, ovine, and caprine farms. Since the mixed teaching farm does not dispose of a milking station, surface floor samples (X3) were taken). Animal management and sample collection were conducted by veterinarians, strictly implementing the national and European guidelines and standards of animal welfare (Real Decreto 53/2013 and European Union Directive 2010/63/EU, respectively). To avoid possible cross‐contamination among sampled animals, faecal samples were obtained by rectal grab and were collected from randomly selected animals during each visit. The approval of the Animal Ethics and Experimentation Committee is not required for this routine veterinary practice. Each sample was collected into a sterile bottle using clean sampling utensils and gloves. Samples from farm environments, such as food troughs (swabs), water troughs (swabs), and bedding samples, were collected from different locations on each farm.
  All solid samples were stored in individual sterile bags. Sample collection was carried out under aseptic conditions, using sterile gloves. Farm environmental samples were collected using disposable gloves or swabs. All samples were stored and transported with ice packs and were processed within 3 to 12 h post‐collection.
- **Natural environments**. Three natural environments with the presence of wild animals were sampled (Albufera Natural Park (freshwater lake), Turia River (river), and the Moro marsh). A total of 456 (*N* = 456) samples were collected. The same number of water (X12), plants (X13), and soil (X13) samples were collected in each area. For water samples, 100 mL were collected for each sample, whereas 8 g of content were collected for plants and soil samples. All samples were stored and transported with ice packs and were processed within 3–12 h post‐collection.
- **Food processing environments**. Isolates of *L. monocytogenes* were obtained during official surveillance in restaurants and food industries from 2019 to 2023 in the same geographical region of southeast Spain (Figure S1). A total of 7395 samples were analysed throughout winter 2019 to winter 2023. Following the Commission Regulation (EC) No 2073/2005, food samples included smoked fish products, ready‐to‐eat meat products, dairy products, prepared dishes, fruits, and vegetable salads. Food processing environmental samples were taken from surfaces (i) in contact with food and (ii) not in contact with food, using abrasive sponges. Since the objective of the European official surveillance is to control the presence of *L. monocytogenes* in food and hospitality following the Commission Regulation (EC) No 2073/2005, only *L. monocytogenes* was isolated and taken into account, regardless of the possible presence of another *Listeria* spp. in the same sample.

### 2.2 *Listeria* spp. Isolation and Identification

Isolation and identification of *L. monocytogenes* in food and food processing environmental samples were conducted according to EN/ISO 11290‐1/2. For this, samples were diluted 1/10 in Half‐Fraser broth (Scharlab, Spain) and incubated at 30°C for 24 h for primary enrichment. Next, 100 mL of the incubated suspension were transferred to 10 mL Fraser broth (Scharlab, Spain) and incubated at 37°C for 24 h. Plating was performed using *Listeria* Chromogenic Agar Base according to Ottaviani and Agosti (ALOA) (Scharlab, Spain), and RAPID'*L.mono* (BioRad, USA) agar plates for EN/ISO 11290‐1 or using ALOA agar plates for EN/ISO 11290‐2. Colonies compatible with *L. monocytogenes* were confirmed by Real‐Time PCR.

For solid material obtained from farm and natural environments, *Listeria* spp. isolation was performed as previously described (Palacios‐Gorba et al. 2021; Quereda et al. 2020). Eight grams were taken from the original sample using a sterilised spatula and were diluted 1/10 in Half‐Fraser broth. Then, samples were homogenised and incubated at 30°C for 24 h for enrichment. Swab samples were placed in 10 mL Half Fraser broth, vortexed for 2 min, and then incubated at 30°C for 24 h for enrichment. For water samples, 100 mL of each sample were divided into 33 mL and deposited in 3 sterile centrifuge tubes and centrifuged for 40 min at 4000 rpm at 21°C. After centrifugation was completed, 30 mL of the supernatant from each tube were removed, and the remaining 3 mL from each tube were added to 81 mL of Half Fraser to complete the 1/10 dilution and incubated at 30°C for 24 h for enrichment. One hundred microlitres of the incubated suspension were transferred to 10 mL Fraser broth and incubated at 37°C for 24 h. After the second enrichment, 100 μL enriched culture and two 10‐fold dilutions were transferred to RAPID'*L.mono* plates and incubated at 37°C for 24 h. Characteristic colonies of *Listeria* spp. in RAPID'*L.mono* plates were white or blue, convex and round, with or without a yellow halo, 1–2 mm [ *L. monocytogenes* (PIPLC +/xylose−) forms blue colonies, whereas other *Listeria* spp. form white colonies]. When more than one compatible colony type was detected on RAPID'*L.mono* plates, one blue colony (compatible with pathogenic *Listeria* spp.) and one white colony (compatible with non‐pathogenic *Listeria* spp.) were selected and picked for further confirmation in selective Oxford agar plates for *Listeria* (Scharlab). Confirmed *Listeria* spp. colonies on Oxford agar plates were grey‐green with a black sunken centre and a black halo, measuring approximately 2 mm in diameter. Confirmed *Listeria* spp. colonies on Columbia CNA agar with 5% sheep blood agar plates were flat and opaque, measuring 1–2 mm in diameter.

All the isolates obtained in the study were preserved in glycerol at −80°C and sent to the World Health Organisation Collaborating Centre for *Listeria* at Institut Pasteur in Paris, France for characterisation, except food and food processing environmental isolates obtained in 2022 and 2023, which were characterised in the Public Health Laboratory of the Dirección General de Salud Pública, Valencia. Species identification was performed using two methods. The first method used matrix‐assisted laser desorption ionisation‐time of flight mass spectrometry (MALDI‐TOF MS) with the MicroFlex LT system and the MBT library DB‐7854 from Bruker Daltonics, Germany (Thouvenot et al. 2018). The second method used WGS (Quereda et al. 2020).

### 2.3 WGS and Assembly

In all isolates obtained from wastewater, ruminant farms, natural environment, and food processing environments from 2019 to 2021, DNA extraction was performed using the NucleoSpin Tissue purification kit from Macherey‐Nagel, Germany. The DNA was extracted from 0.9 mL Brain heart infusion (Difco, USA) cultures that were grown overnight at 35°C. DNA libraries were prepared using the Nextera XT DNA Sample kit from Illumina, USA. The prepared libraries were then sequenced using a NextSeq 500 platform (Illumina), with 2 × 150 bp runs, following the manufacturer's protocol.

For isolates obtained from food and food processing environments obtained in 2022–2023, DNA extraction was performed using an automated method (NucliSens easyMAG, BioMérieux, France) after growth at 37°C for 24 h in Columbia Blood Agar. DNA libraries were prepared using the Nextera XT DNA Sample kit from Illumina, USA. The prepared libraries were then sequenced using a MiSeq platform (Illumina) with 2 × 300 bp runs, following the manufacturer's protocol.

To process the raw sequencing reads of all isolates, the fqCleaner v.3.0 software (https://gitlab.pasteur.fr/GIPhy/fqCleanER) was used to remove low‐quality bases and adapter sequences from raw reads. The trimming steps were performed as previously described by Palacios‐Gorba et al. (2021), Quereda et al. (2020). The trimmed reads were assembled using SPAdes v.3.12.0 (Prjibelski et al. 2020). Automatic k‐mer selection was used during the assembly process, along with the “‐only‐assembler” and “‐careful” options, which optimise the assembly parameters to improve the accuracy and quality of the resulting assembly.

### 2.4 Molecular Typing and Phylogenetic Analysis

*In silico* typing was conducted from the assemblies in BIGSdb‐*Listeria* v.1.30 https://bigsdb.pasteur.fr/listeria; (Moura et al. 2016; Jolley and Maiden 2010), using the BLASTN algorithm and a minimum nucleotide identity and alignment length coverage of 70% and a word size of 10 (Moura et al. 2016). Schemes used included genoserogrouping (Doumith et al. 2004), Multi‐Locus Sequence Typing (MLST; seven loci (Ragon et al. 2008)), core genome MLST (cgMLST; 1748 loci (Moura et al. 2016)), as well as schemes for resistance and virulence genes (244 loci). MLST profiles were classified into sequence types (STs) and grouped into clonal complexes (CCs) as described by Ragon et al. (2008). cgMLST profiles were analysed and categorised into cgMLST types (CTs) and sublineages (SLs) using specific cutoffs (7 and 150 allelic mismatches, respectively, as previously described (Moura et al. 2016)). Minimum spanning trees and single linkage dendrograms were constructed using cgMLST profiles using Bionumerics 7.6 software from Applied Maths (Belgium). The resulting trees and dendrograms were then annotated using iTol v.4.2 software (Letunic and Bork 2021).

### 2.5 Statistical Analysis

Shannon diversity indices and Hutcheson *t*‐test (Hutcheson 1970) were calculated using the web https://www.dataanalytics.org.uk/comparing‐diversity/. The rest of the statistical analyses were performed with GraphPad Prism V8 software system. Chi‐square tests were performed to assess *L. monocytogenes* prevalences and the effect of season. Fisher's exact test was performed to determine the association of lineages with origins. The significance levels were *, p < 0.05; **, p < 0.01; ***, *p* < 0.001; ****, *p* < 0.0001.

## 3 Results

### 3.1 Prevalence of *Listeria* spp. in Wastewater, Ruminant Farms, Natural Environments, and Food Processing Environments

**FIGURE 1:**
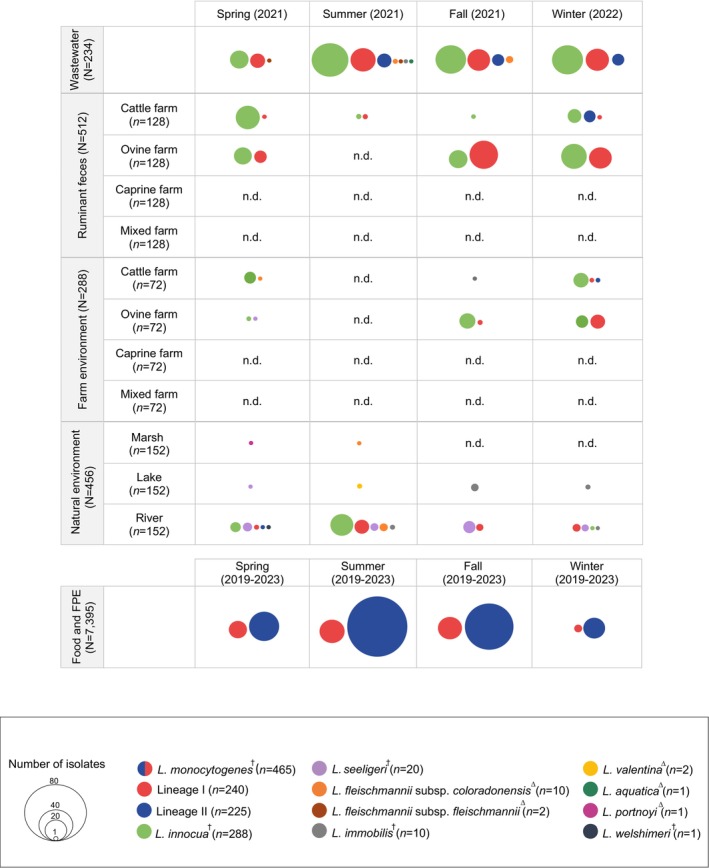
Number of *Listeria* spp. isolates obtained in this study from wastewater (*n* = 234 samples from 4 sites), ruminant faeces (*n* = 512 from 4 farms), ruminant farm environments (*n* = 288 from 4 farms), natural environments (*n* = 456 from 3 sites), and food processing environments (FPE) (*N* = 7395 from regional food surveillance). *Listeria* spp. isolated from wastewater, ruminant faeces, ruminant farms, and natural environments were sampled during four consecutive seasons (spring 2021 to winter 2022). *L. monocytogenes* isolated from food and food processing environments were sampled during four consecutive years (spring 2019 to winter 2023). In the food and food processing environments category, the prevalence of other species than *L. monocytogenes* could not be assessed, since these isolates were obtained from public health authorities who exclusively surveyed *L. monocytogenes* , disregarding the possible presence of other *Listeria* spp. Circle size is proportional to the number of isolates. † *Listeria sensu stricto*; ∆ *Listeria sensu lato*.

We assessed the prevalence of *Listeria* spp. in different environments in Spain from March 2021 to March 2022 during four consecutive seasons (Figures 1 and S1, Table S2). A total of 1490 samples were collected and analysed from: untreated influents of four urban wastewater treatment plants (*n* = 234), four ruminant farms including ruminant faeces (*n* = 512) and farm environment (*n* = 288), and three different natural environments (*n* = 456) (Figure 1). Food and food processing environment samples were analysed based on official surveillance between January 2019 and March 2023 (*n* = 7395).

*Listeria* spp. were detected in all sampled environments; 100% (4/4) of wastewater treatment plants, 50% (2/4) of ruminant farms, 100% of natural environments (3/3) and in all sampling seasons (Figure 1). The overall prevalence of *Listeria* spp. was 36.6% (545/1490), of which 529 isolates belonged to *Listeria sensu stricto* (97.1%, 529/545) and 16 to *Listeria sensu lato* (2.9%, 16/545) (Figure 1, Table S3). The prevalence varied significantly between the sampled environments, from 14.9% to 64.1%, and was overall higher in wastewater (64.1%, 150/234) than in ruminant faeces (23.6%, 121/512), farm environment (14.9%, 43/288), and natural environment (16.2%, 74/456) (Figure 1, Table S2).

Among all isolates, the most common *Listeria* spp. were *L. innocua* (52.8%; 288/545) and *L. monocytogenes* (38.5%, 210/545) (Figure 1, Table S3). Co‐occurrence of the two *Listeria* spp. was detected in 38.8% (91/234), 8% (41/512), 4.5% (13/288), and 1.3% (6/456) of wastewater, ruminant faeces, farm environment, and natural environment samples, respectively. Co‐occurrence of *L. seeligeri* and *L. monocytogenes* was detected in one natural environment sample (0.2%, 1/456) (Table S2).

The prevalence of *L. innocua* was 56.8% (133/234) in wastewater, 17.2% (88/512) in ruminant faeces, 13.9% (40/288) in farm environment, and 5.9% (27/456) in natural environment samples, whereas the prevalence of *L. monocytogenes* was 42.7% (100/234), 14.6% (75/512), 5.6% (16/288), and 4.2% (19/456), respectively (Figure 1, Table S3).

### 3.2 *L. monocytogenes* From Wastewater, Ruminant Farms, and Natural Environment Belong Preferentially to Lineage I

A total of 800 *Listeria* spp. isolates were obtained, 545 from wastewater, ruminant farms, and natural environments (68.1%, 545/800); 255 from food and food processing environments (31.9%, 255/800). In the latter, only *L. monocytogenes* was obtained, since only this species was analysed during official surveillance. For this reason, *L. monocytogenes* food isolates were considered separately from the rest of the sampled niches in further analysis. All isolates were characterised at the genomic level (Figure 1, Tables S2 and S3).

Two hundred and forty‐two (44.4%, 242/545), 163 (29.9%, 163/545), 59 (10.8%, 59/545), and 81 (14.9%, 81/545) *Listeria* spp. isolates were obtained from wastewater samples, ruminant faeces, farm environment, and natural environment, respectively (Figure 1, Tables S2 and S3). Nine *Listeria* spp. were identified: *L. innocua* (52.8%, 288/545), *L. monocytogenes* (38.5%, 210/545), *L. seeligeri* (3.7%, 20/545), *L. fleischmannii* subsp. *coloradonensis* (1.8%, 10/545), *L. fleischmannii* subsp. *fleischmannii* (0.4%, 2/545), *L. immobilis* (1.8%, 10/545), *L. valentina* (0.4%, 2/545), *L. aquatica* (0.2%, 1/545), *L. portnoyi* (0.2%, 1/545), and *L. welshimeri* (0.2%, 1/545) (Figure 1, Tables S2 and S3).

**FIGURE 2:**
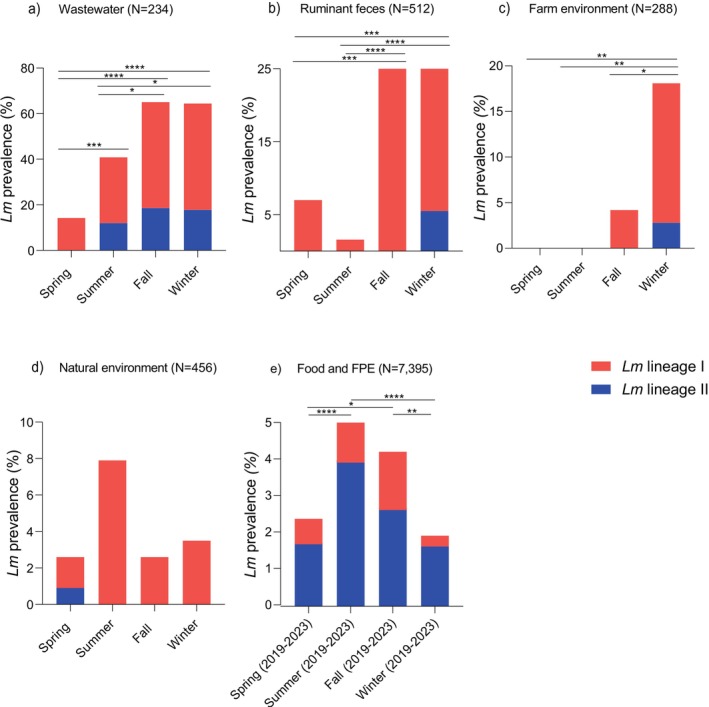
Prevalence of *L. monocytogenes* positive samples among wastewater, ruminant faeces, farm environment, natural environment, and food processing environments (FPE). *L. monocytogenes* prevalence in wastewater, ruminant farms, and natural environment was assessed from spring 2021 to winter 2022; whereas *L. monocytogenes* prevalence among food and food processing environments was assessed from spring 2019 to winter 2023. *N* indicates the number of analysed samples.

**FIGURE 3:**
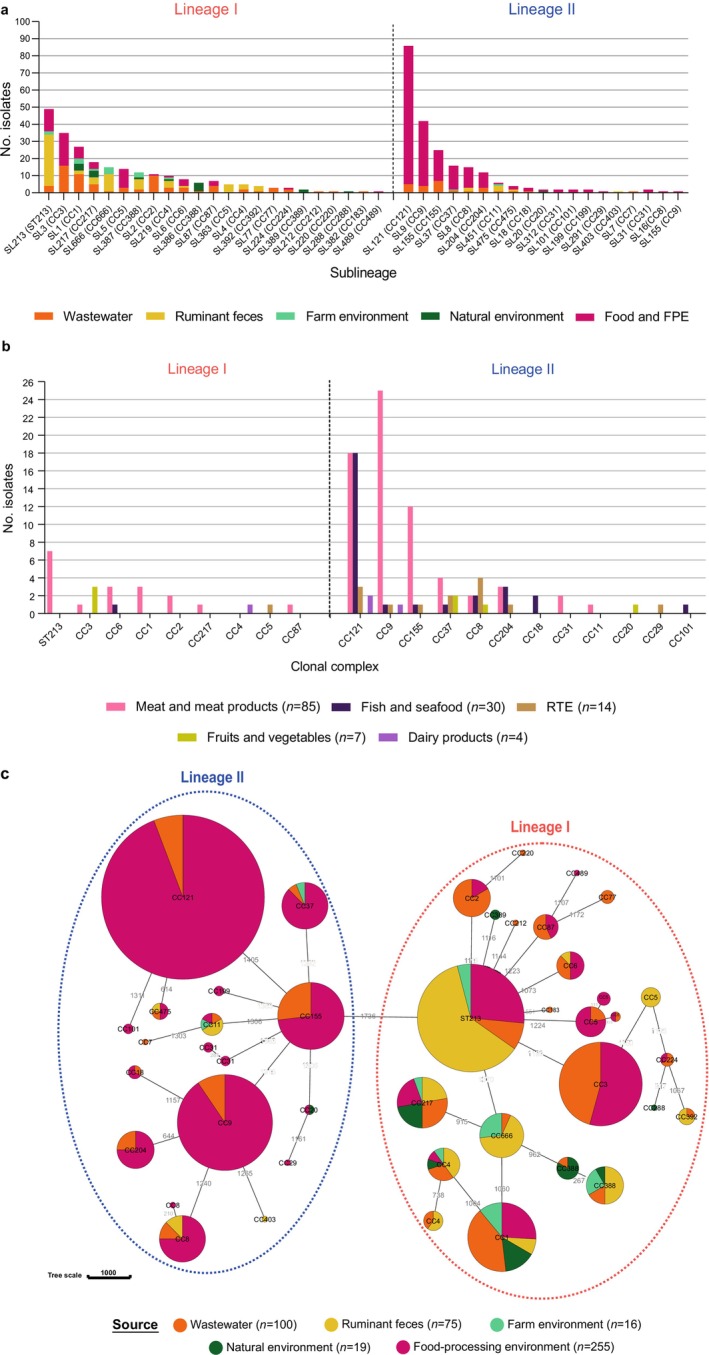
(a) Diversity of *L. monocytogenes* isolates collected in this study (*N* = 465) based on cgMLST (1748 loci) analyses. Distribution of sublineages (SLs) in wastewater (*n* = 100), ruminant faeces (*n* = 75), farm environment (*n* = 16), natural environment (*n* = 19), and food processing environments (FPE) (*n* = 255) *L. monocytogenes* isolates. Corresponding clonal complexes (CCs), defined on the basis of seven‐locus MLST, are shown in brackets. (b) Distribution of CCs from the five major food‐product categories of this study. (c) Minimum spanning tree based on the cgMLST profiles *L. monocytogenes* observed in each sampled ecological niche in this study. Circle sizes are proportional to the number of isolates and are coloured by source type, as in panel (a). CCs, defined on the basis of seven‐locus MLST, are shown in each circle. Dashed lines delimitate lineages and are coloured according to the phylogenetic lineage (red, lineage I; blue, lineage II). Values shown in connecting lines denote the number of allelic differences between profiles.

*L. monocytogenes* from wastewater, ruminant farms, and the natural environment belonged to lineages I (82.8%. 174/210; serogroups IVb, *n* = 135; IVb‐v1, *n* = 1; and IIb, *n* = 38) and II (17.1%, 36/210; serogroups IIa, *n* = 32 and IIc, *n* = 4) (Figure 2, Tables S2 and S3). Among *L. monocytogenes* isolates obtained from food and food processing environments (*n* = 255), 25.9% belonged to lineage I (66/255; serogroups IIb, *n* = 35; and IVb, *n* = 31) and 74.1% to lineage II (189/255; serogroups IIa, *n* = 151; IIc, *n* = 38) (Figure 2, Tables S2 and S3). Considering all niches, the most prevalent sublineages (SLs) and CCs among *L. monocytogenes* lineage I were SL213/ST213, SL3/CC3, and SL1/CC1 (Figure 3a–c, Table S2). The most common SLs and CCs among *L. monocytogenes* lineage II recovered were SL121/CC121, SL9/CC9, and SL155/CC155 (Figure 3a–c, Table S2). *L. monocytogenes* from wastewater, ruminant farms, and the natural environment were mainly associated with lineage I (*p* < 0.01), whereas *L. monocytogenes* from food and food processing environments belonged preferentially to lineage II (*p* < 0.0001).

Two hundred and fifty‐five different CTs were detected: 231 (90.6%) unique to this study and 24 (9.4%) previously reported in BIGSdb‐*Listeria* (Tables S2 and S4). The majority of detected CTs included only one isolate (69.4%, 177/255), whereas 78 (30.6%, 78/255) included 2–45 isolates (Tables S2 and S5). Up to 32 (12.5%), 21 (8.2%), 11 (4.3%), 5 (2%), and 16 (6.3%) different CTs could be isolated per season from wastewater, ruminant faeces, ruminant farm environment, natural environment, and food processing environment, respectively, from March 2021 to March 2022 (Table S2). Considering this time frame, a higher CT diversity was detected in wastewater, ruminant farm, and natural environment samples (129 CTs obtained from 1490 samples) than in food and food processing environments (34 CTs obtained from 1715 samples) (Shannon indexes 4.40 vs. 3.43; Hutcheson *t*‐test, *p* < 0.0001). Eleven CTs (4.3%, 11/255) were common to both sets of samples.

### 3.3 Population Structure of *L. monocytogenes* Across Food Categories

Among *L. monocytogenes* isolates obtained from food‐associated environments (*n* = 255), 140 isolates were obtained from different foods (*n* = 140) and 115 from food processing environments (*n* = 115) (Table S2). *L. monocytogenes* was detected among meat and meat products (60.7%, 85/140), fish and seafood (21.4%, 30/140), mixed ready‐to‐eat (RTE) products (10%, 14/140), fruits and vegetables (5%, 7/140), and dairy products (2.8%, 4/140) (Table S2). Due to the low number of isolates detected in the RTE, fruits and vegetables, and dairy products, comparison of CCs and STs was only performed between meat and meat products and fish and seafood categories. The most frequent CCs detected within the meat and meat products category were CC9 (29.4%, 25/85), CC121 (21.2%, 18/85), CC155 (14.1%, 12/85), and ST213 (8.2%, 7/85) (Figure 3b, Table S2). The most frequent CC detected within the fish and seafood category was CC121 (60%, 18/30) (Figure 3b, Table S2). While CC121 was isolated from meat and meat products, as well as fish and seafood, CC9, CC155, and ST213 were mainly isolated from meat and meat products.

### 3.4 Persistence and Circulation of *L. monocytogenes* Between Different Environments

Thirteen CTs were isolated in more than one niche and season: 10 CTs were detected in wastewater and food processing environments (e.g., L2‐SL9‐ST9‐CT7203 was detected in food and food processing environment samples from summer 2019 to fall 2023 and in wastewater samples in fall 2021). L1‐SL1‐ST1‐CT2060 was detected in wastewater (fall 2021) and farm environment (winter 2022). L1‐SL213‐ST213‐CT1846 was detected in food and food processing environment samples (from fall 2019 to fall 2023), wastewater (summer 2021, fall 2021), and ruminant farms (fall 2021, winter 2022) (Tables S2, S5, and S6). Interestingly, no CTs isolated from natural environments were detected in other sampled niches. Five CTs of lineage I (2.1%, 5/240) and 19 CTs of lineage II (8.4%, 19/225) isolated in this study were identical to those detected in other reports worldwide (Table S4).

*L. monocytogenes* genotypes with a continuous presence were defined as persistent (Stasiewicz et al. 2015). Twenty‐six persistent *L. monocytogenes* CTs were identified in food processing environments (9.4%, 24/255) or sheep farms (0.8%, 2/255) (Tables S2, S5, and S6). For example, L1‐SL3‐ST3‐CT7208 was detected in the food processing environment of company 2 in spring 2019, spring 2021, and spring 2023. One hundred and thirty‐nine *L. monocytogenes* isolates from food processing environments and farm environments belonged to these 26 persistent CTs.

Moreover, CTs that persisted in more than one food processing facility were detected. For example, L1‐ST213‐CT1846 was identified in Company 26 (which processes meat products) during fall 2019 and fall 2020, and was subsequently detected in Company 5 (dedicated to meat product production) during fall 2020 and fall 2023 (Table S6). Similarly, L2‐CC121‐CT903 was found in Company 18 (dedicated to fish products) in fall 2020 and spring 2021, and later in Company 19 (which elaborates RTE products), during summer 2022 and spring 2023 (Table S6). Another case involved L2‐CC204‐CT11448, which was isolated from Company 24 (engaged in meat product elaboration) in spring and summer 2021, and from Company 25 (which processes salmon products) in both summer and winter 2023 (Table S6).

These results highlight that (i) the same *L. monocytogenes* CTs circulate between wastewater, domestic ruminant farms, and food processing environments in the same geographic region, and between countries, and (ii) that *L. monocytogenes* can persist in food processing environments and ruminant farms.

### 3.5 *L. monocytogenes* From Different Environments Have Distinct Virulence and Resistance Determinants

**FIGURE 4:**
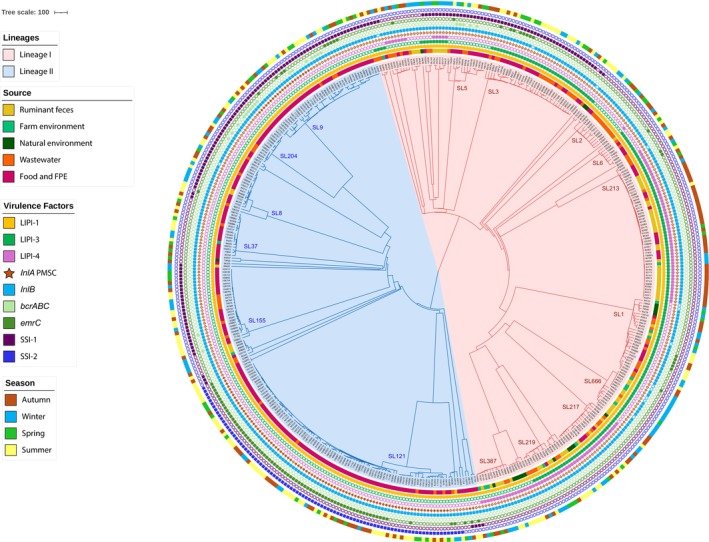
Genetic diversity of 465 *L. monocytogenes* isolates sequenced in this study. cgMLST single linkage dendrogram. Branches are coloured by phylogenetic lineage (LI, red; LII, blue). SLs with more than 10 isolates are labelled in the tree. The inner and outer rings show the isolate's source and season, respectively, according to the colour codes shown on the left. Colour‐filled dark boxes indicate the presence of selected genetic traits: *Listeria* pathogenic islands (LIPI‐1, LIPI‐3, and LIPI‐4), internalin (*inlB*), benzalkonium chloride (*bcrABC*, *emrC*), pH or oxidative stress (SSI‐1, SSI‐2). Brown‐filled stars represent genes with truncations leading to Internalin A (*inlA*) premature stop codons (PMSCs).

**FIGURE 5:**
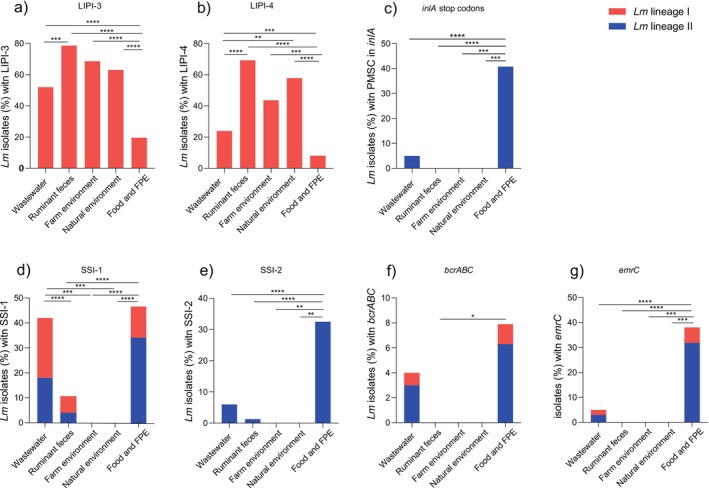
Prevalence of genetic traits involved in virulence and acquired resistance towards disinfectants. *L. monocytogenes* isolates were obtained from wastewater (*n* = 100), ruminant faeces (*n* = 75), farm environment (*n* = 16), natural environment (*n* = 19), and food processing environments (FPE) (*n* = 255). *Listeria* pathogenic islands (LIPI‐3 and LIPI‐4), acquired premature stop codons (PMSCs) in *inlA*, SSI‐1, SSI‐2, and acquired resistance loci towards benzalkonium chloride (*bcrABC* and *emrC*) are shown. Statistically significant associations (Fisher's exact test) are indicated with asterisks. *p ≤ 0.05, **p ≤ 0.01, ***p ≤ 0.001, ****p ≤ 0.0001.

All *L. monocytogenes* isolates carried LIPI‐1, which is part of its core genome (Figure 4). LIPI‐3 and LIPI‐4 associated with hypervirulence (Maury et al. 2016) were significantly more abundant in wastewater, ruminant faeces, and farm and natural environments than in food and food processing environments (*p* < 0.001) (Figures 4 and 5a,b, Table S2). Premature stop codons (PMSCs) in *inlA* were detected only in *L. monocytogenes* lineage II isolates and were more frequent in isolates from food and food processing environments (40.4%, 103/255) than in the other sampled environments (prevalences from 0% to 5%) (*p* < 0.001) (Figures 4 and 5c, Table S2).

SSI‐1 (tolerance to high salt and low pH) was detected more frequently in food and food processing environments and wastewater than in the rest of the sampled environments (*p* < 0.001) (Figures 4 and 5d). The SSI‐2 (encoding tolerance to oxidative stress and alkaline conditions), *bcrABC* and *emrC* genes were more frequently present in isolates from food and food processing environments than in the rest of the sampled environments (*p* < 0.05) (Figures 4 and 5e–g, Table S2).

### 3.6 *Listeria* spp. Are More Prevalent in Host‐Derived Environments Than in Natural Environments

**FIGURE 6:**
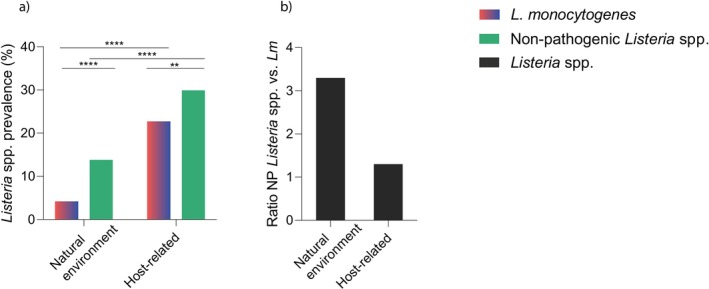
(a) Prevalence of *L. monocytogenes* (*Lm*) and non‐pathogenic (NP) *Listeria* spp. positive samples among natural environment (*n* = 81) and host‐related *Listeria* spp. isolates (*n* = 405). Host‐related category (*N* = 746) contained samples from wastewater (*n* = 234) and ruminant faeces (*n* = 512). *Listeria* spp. prevalence in natural environmental samples (*N* = 456) and in host‐related samples (*N* = 746) was assessed from spring 2021 to winter 2022. Statistically significant associations (Fisher's exact test) of *Listeria* spp. with origin ‘Natural environments’ or ‘Host‐related’) are indicated with asterisks. **p ≤ 0.01, ****p ≤ 0.0001. (b) Ratio of non‐pathogenic (NP) *Listeria* spp. versus *L. monocytogenes* (*Lm*) isolates in natural environment and host‐related environments.

The prevalence of non‐pathogenic *Listeria* spp. and *L. monocytogenes* was higher in host‐derived samples (wastewater and ruminant faeces) (30.8%, 230/746; 23.5%, 175/746, respectively) than in natural environment samples (rivers, lakes, marshes) (13.6%, 62/456; 4.2%, 19/456, respectively) (*p* < 0.0001) (Figure 6a).

In natural and host‐derived environments, the overall prevalence of non‐pathogenic *Listeria* spp. (13.6% and 30.8%, respectively) was higher than the prevalence of *L. monocytogenes* (4.2%, and 23.4%, respectively) (*p* < 0.0001 and *p* < 0.01, respectively) (Figure 6a). The ratio of non‐pathogenic *Listeria* spp. versus *L. monocytogenes* was higher in natural environments than in host‐derived environments (3.3 vs. 1.3) (Figure 6b).

Taken together, these results show that (i) *Listeria* spp. were more prevalent in host‐derived environments than in natural environments and (ii) that the prevalence of non‐pathogenic *Listeria* spp. was higher than that of *L. monocytogenes* in these niches.

### 3.7 Seasonal Influence on the Prevalence of *L. monocytogenes* Depends on the Type of Environment

The presence of *L. monocytogenes* in all seasons was detected only in wastewater and food processing samples (Figures 1, 2, and 4). In food and food processing environments, the overall prevalence of *L. monocytogenes* was higher in summer and fall (*p* < 0.05). In ruminant faeces, farm environment, and wastewater, the overall prevalence of *L. monocytogenes* was higher in fall and winter (*p* < 0.05) (Figures 2 and 4). In natural environments, no significant differences were observed between seasons (*p* > 0.05) (Figures 2 and 4).

## 4 Discussion

Understanding the environmental preferences and circulation of *L. monocytogenes* between the natural environment, ruminants, farms, food, and human hosts remains a key missing piece to fully unravel the epidemiology of this bacterium and its association with different ecological niches. This has important public health implications, as it will help to develop more specific control measures and could help to prevent the infection of ruminants and humans. To the best of our knowledge, this is the most comprehensive study of the genomic characteristics, ecology, and prevalence of *Listeria* spp. in diverse and interconnected environments. Previous studies have analysed the environmental distribution of *Listeria* spp. across different environments but have not addressed the genetic diversity of *L. monocytogenes* (MacGowan et al. 1994; Chapin et al. 2014). Subsequent research has investigated the genetic diversity of *L. monocytogenes* using MLST (Sauders et al. 2006; Linke et al. 2014; Haase et al. 2014) and cgMLST (Palacios‐Gorba et al. 2021; Raschle et al. 2021; Liao et al. 2023) in different environments. While some studies focused exclusively on *L. monocytogenes* and overlooked other *Listeria* spp. (Sauders et al. 2006; Haase et al. 2014; Raschle et al. 2021), other large‐scale studies limited their scope to a narrower range of environmental niches (Palacios‐Gorba et al. 2021; Linke et al. 2014; Liao et al. 2023). The present study demonstrates that *L. monocytogenes* circulating in urban wastewater and ruminant farms preferentially belongs to lineage I and harbours virulence‐associated traits (e.g., intact InlA, LIPI‐3, and LIPI‐4), as do *L. monocytogenes* isolated from human and animal clinical cases (Palacios‐Gorba et al. 2021; Maury et al. 2016, 2019; Dreyer et al. 2016).

Previous publications have suggested that *Listeria* spp. are ubiquitous bacteria whose primary habitat is soil and decaying vegetation (Linke et al. 2014; Welshimer and Donker‐Voet 1971; Weis and Seeliger 1975; Vivant et al. 2013). Our data show that *Listeria* spp. are more abundant in host‐associated environments than in natural environments and that non‐pathogenic *Listeria* spp. are more prevalent than *L. monocytogenes* in these niches. Our results are in agreement with a previous study that analysed risk factors associated with *L. monocytogenes* contamination of produce fields (Strawn et al. 2013). Manure application, reported observation of wildlife within 3 days before sample collection, and recent worker activity (within 3 days prior to sample collection) had higher odds of *L. monocytogenes* isolation than fields where these activities were not reported (Strawn et al. 2013). Similarly, another study that analysed the diversity of *Listeria* species in urban and natural environments concluded that *L. monocytogenes* and *L. innocua* were overrepresented among isolates from urban environments (Sauders et al. 2012).

Taken together, our data suggest the existence of a cycle in which anthropogenic activities and ruminants are the ecological drivers of the spread of *Listeria* spp. The environmental distribution of *L. monocytogenes* is related to virulence: although the human population is mainly exposed to the hypovirulent lineage II due to its higher prevalence in food and food processing environments, the hypervirulent lineage I is mostly found in urban wastewater, which may reflect the better adaptation of lineage I to colonise and replicate in the intestinal lumen of human hosts, as previously shown (Hafner et al. 2024; Maury et al. 2019). This finding, together with the fact that (i) lineage I is overrepresented in faecal samples from dairy ruminants (present study and (Palacios‐Gorba et al. 2021)); (ii) human and ruminant listeriosis cases are mainly caused by *L. monocytogenes* lineage I (Maury et al. 2016; Bagatella et al. 2022); (iii) the phylogeography of the hypervirulent *L. monocytogenes* CC1 is linked to global cattle trade, and CC1 is also locally established in the farm environment (Palacios‐Gorba et al. 2021; Moura et al. 2021); (iv) *L. monocytogenes* is detected in 10% and up to 43.3% of asymptomatic human and dairy ruminant stool samples, respectively (Hafner et al. 2021; Palacios‐Gorba et al. 2021); (v) clinically overt *L. monocytogenes* infection is rare and is not involved in direct horizontal transmission between humans and between animals (de Graaf et al. 2017); and (vi) lineage II forms more biofilms and has acquired resistance genes to disinfectants (Maury et al. 2019), suggest that *L. monocytogenes* lineages I and II represent two distinct populations influenced by different ecological constraints, with lineage I *L. monocytogenes* having established a preferential association with human and animal hosts (Figure S2) (Disson et al. 2021; Hafner et al. 2024; Maury et al. 2019). Our results also show significant differences in the geographic spread patterns between *L. monocytogenes* lineage I and lineage II strains. However, it is important to note that a few dominant STs drive the genomic distribution patterns observed within each lineage in the present study and may not reflect the characteristics of all STs comprising the entire lineage. Similar to our data, culture‐based studies have shown that *L. monocytogenes* and *L. innocua* are the most prevalent *Listeria* spp. in foods, food processing environments, wastewater, urban and ruminant farm environments (Palacios‐Gorba et al. 2021; MacGowan et al. 1994; Sauders et al. 2012). The higher detection of *L. innocua* versus *L. monocytogenes* in the present and previous studies should be interpreted with caution since the presence of *L. innocua* could mask the detection of *L. monocytogenes* due to competitive growth during enrichment protocols (Petran and Swanson 1993). In contrast, metagenomic approaches comparing the relative abundance (proportion of a species in a given sample) have reported a higher relative abundance of *L. monocytogenes* in soil and host samples (Hafner et al. 2021). In previous studies of natural environment samples, *L. seeligeri* and *L. monocytogenes* (Frances et al. 1991), or *L. seeligeri* and *L. welshimeri* (Sauders et al. 2012) were the most prevalent species. Accordingly, in our study, 95% (19/20) of the *L. seeligeri* isolates were isolated from natural environments. Collectively, these data suggest that although *Listeria* is a widespread genus, different *Listeria* spp. have unique ecological preferences.

The sources of entry of *L. monocytogenes* into the food chain are not fully understood. In the current study, 5.1% (13/255) of the detected CTs were isolated in more than one sampled niche and season, demonstrating the circulation of *L. monocytogenes* through different environmental niches. In addition, 26 *L. monocytogenes* CTs were persistent in different environments such as the food industry and ruminant farms, which could lead to recurrent food contamination or infection.

The most frequently identified CCs and ST in meat products in the present study were CC9, CC121, CC155, and ST213. The high frequency of these CCs in meat has been previously reported in France (CC9 and CC121) (Maury et al. 2019), China (CC9 and CC121) (Zhang et al. 2025), and Korea (CC9 and CC155) (Hong et al. 2025). CC121 was also associated with fish and fish products in the present study, a finding also reported in France (Maury et al. 2019) and China (Zhang et al. 2025). Regarding ST213, few studies in the literature have reported its isolation. ST213 was detected in Mexico in 2018, associated with fresh produce (Gómez‐Baltazar et al. 2025) and reported in the BIGSdb‐*Listeria* database from six human clinical isolates originating from Spain (2001, 2011, and 2015) and the United States (2005 and 2019). The higher overall prevalence of *L. monocytogenes* during summer and fall in food and food processing environments is consistent with the seasonality of the confirmed human cases of listeriosis in the European Union (EFSA 2023). Furthermore, the increased prevalence observed in ruminant faeces, farm environments, and wastewater during fall and winter is consistent with previous studies reporting higher faecal carriage of *L. monocytogenes* in colder seasons (Palacios‐Gorba et al. 2021; Castro et al. 2018).

## 5 Conclusion

In conclusion, our data show that (i) *L. innocua* , followed by *L. monocytogenes* , were the most prevalent *Listeria* spp. in wastewater, ruminant faeces, ruminant farms, and natural environments; (ii) co‐occurrence of *L. innocua* and *L. monocytogenes* was observed in 10.1% of isolates obtained from wastewater, ruminant faeces, ruminant farms, and natural environments; (iii) whereas *L. monocytogenes* lineage II was predominant in food and food processing environments, lineage I was highly predominant in wastewater, ruminant faeces, ruminant farms, and natural environments; (iv) *L. monocytogenes* was able to persist in food processing environments for at least 4 years and in ruminant farms for at least two consecutive seasons; (v) evidenced circulation of CTs between different environments was demonstrated by WGS; (vi) acquired resistance traits towards disinfectants were more frequent among food and food processing environments than in other sampled niches; (vii) LIPI‐3, LIPI‐4, and intact InlA were more frequent among *L. monocytogenes* isolates from wastewater, ruminant farms, and natural environments than in food and food processing environments; (viii) *Listeria* spp. were more prevalent in host‐associated environments than in natural environments; (ix) the prevalence of non‐pathogenic *Listeria* spp. was higher than that of *L. monocytogenes* in natural environments when culture‐based approaches were used; and (x) the overall prevalence of *L. monocytogenes* was higher in summer and fall in food and food processing environments and higher in fall and winter in ruminant faeces, farm environments, and wastewater.

Future insights into the ecology of *Listeria* spp. require further investigation into their distribution in diverse environments. This will facilitate the identification of distinct ecological niches and reservoirs of *Listeria* spp., as well as the transmission pathways of pathogenic *Listeria*. This study improves the understanding of the prevalence and ecology of *Listeria* spp. in various environments and hosts and could therefore provide valuable insights into the prevention and management of listeriosis in humans and animals.

## Author Contributions

**Yuval Markovich:** writing – review and editing, writing – original draft, visualization, validation, resources, methodology, investigation, formal analysis. **Alexandra Moura:** investigation, methodology, writing – review and editing, data curation. **Jesús Gomis:** resources, investigation, methodology. **Alexandre Leclercq:** methodology, investigation, data curation. **Ángel Gómez‐Martín:** investigation, resources, methodology. **Hélène Bracq‐Dieye:** investigation. **Carla Palacios‐Gorba:** writing – review and editing, formal analysis, data curation, validation, investigation. **Nathalie Tessaud‐Rita:** investigation. **Susana Ortolá:** investigation, data curation, resources, methodology. **Guillaume Vales:** investigation. **M‐Adela Yáñez:** resources, methodology, investigation. **Pierre Thouvenot:** investigation. **Philippe Pérot:** data curation, writing – review and editing, investigation, methodology. **Marc Lecuit:** writing – review and editing, funding acquisition, investigation, methodology, data curation, resources, supervision. **Juan J. Quereda:** conceptualization, funding acquisition, writing – original draft, writing – review and editing, visualization, validation, supervision, project administration, methodology, formal analysis, investigation, resources.

## Disclosure

The authors have nothing to report.

## Conflicts of Interest

The authors declare no conflicts of interest.

## Supporting information


**Figure S1:** Geographic map of Spain showing the distribution of the different sampling sites.
**Figure S2:** Host‐associated versus saprophytic lifestyles of *L. monocytogenes* . *L. monocytogenes* lineages I and II represent two distinct populations that likely evolve under different ecological constraints. In our sampling study, lineage I *L. monocytogenes* strains appear to cycle between ruminants, their environment, and urban wastewater. However, lineage II *L. monocytogenes* strains are predominantly associated with food and food processing environments and appear to have a lower host association capacity. Host‐adapted *L. monocytogenes* strains predominantly belong to hypervirulent clonal complexes and are associated with faecal shedding, they harbour LIPI‐3 (and LIPI‐4 in CC4), functional InlA and high SigB responsiveness (Jacquet et al. 2004; Quereda et al. 2016; Maury et al. 2016, 2019; Palacios‐Gorba et al. 2021; Hafner et al. 2021, 2024). In contrast, saprophytically adapted strains are predominantly hypovirulent, express a truncated form of InlA, SSI‐1 and SSI‐2, harbour disinfectant resistance genes (*bcrABC*, *emrC*), exhibit low SigB responsiveness, and form biofilm (Maury et al. 2016, 2019; Hafner et al. 2024).

**Table S1:** Characteristics and management practices of the investigated wastewater treatment plants and dairy farms.
**Table S2:** *Listeria* isolates characterised in this study (*N* = 800).
**Table S3:** Number of *Listeria* spp. isolates (%) obtained in this study (*N* = 800).
**Table S4:** cgMLST types previously defined at BIGsdb‐*Listeria* detected in this study (*n =* 24).
**Table S5:** *L. monocytogenes* cgMLST types detected comprising two or more isolates (*n* = 78 types out of 255).
**Table S6:** *L. monocytogenes* repetitive clonal types (CTs) in different sampled niches and over consecutive periods. Isolates from food and food processing environments were obtained from 2019 to 2023, whereas isolates from ruminant faeces, and farm environments were obtained from March 2021 to March 2022. Each number (1 to 69) represents a specific industry or hospitality. Capital O represents the ovine farm.

## Data Availability

Sequences obtained in this study are publicly available at the European Nucleotide Archive (BioProject PRJEB85267) and BIGSdb‐*Listeria* (bigsdb.pasteur.fr/listeria).
