# Supplementary material for: Predominance of L. monocytogenes Lineage I Clones in Wastewater, Ruminants, and Natural Environments
Source: Environ Microbiol. 2025 Sep 7;27(9):e70169. doi: 10.1111/1462-2920.70169 (PMC12415317; doi:10.1111/1462-2920.70169)
Supplement: Supplementary file 1 — Figure S1: Geographic map of Spain showing the distribution of the different sampling sites. Figure S2: Host‐associated versus saprophytic lifestyles of L. monocytogenes . L. monocytogenes lineages I and II represent two distinct populations that likely evolve under different ecological constraints. In our sampling study, lineage I L. monocytogenes strains appear to cycle between ruminants, their environment, and urban wastewater. However, lineage II L. monocytogenes strains are predominantly associated with food and food processing environments and appear to have a lower host association capacity. Host‐adapted L. monocytogenes strains predominantly belong to hypervirulent clonal complexes and are associated with faecal shedding, they harbour LIPI‐3 (and LIPI‐4 in CC4), functional InlA and high SigB responsiveness (Jacquet et al. 2004; Quereda et al. 2016; Maury et al. 2016, 2019; Palacios‐Gorba et al. 2021; Hafner et al. 2021, 2024). In contrast, saprophytically adapted strains are predominantly hypovirulent, express a truncated form of InlA, SSI‐1 and SSI‐2, harbour disinfectant resistance genes (bcrABC, emrC), exhibit low SigB responsiveness, and form biofilm (Maury et al. 2016, 2019; Hafner et al. 2024). [file EMI-27-e70169-s001.pdf]

Figure S1. Geographic map of Spain showing the distribution of the different sampling sites.

Figure S2. Host-associated versus saprophytic lifestyles of *L. monocytogenes*. *L. monocytogenes* lineages I and II represent two distinct populations that likely evolve under different ecological constraints. In our sampling study, lineage I *L. monocytogenes* strains appear to cycle between cattle, their environment, and urban wastewater. However, lineage II *L. monocytogenes* strains are predominantly associated with food and food processing environments and appear to have a lower host association capacity. Host-adapted *L. monocytogenes* strains predominantly belong to hypervirulent clonal complexes and are associated with fecal shedding, they harbor LIPI-3 (and LIPI-4 in CC4), functional InlA and high SigB responsiveness (Jacquet et al., 2004; Quereda et al., 2016; Maury et al., 2016, 2019; Palacios-Gorba et al., 2021; Hafner et al., 2021, 2024). In contrast, saprophytically adapted strains are predominantly hypovirulent, express a truncated form of InlA, SSI-1 and SSI-2, harbor disinfectant resistance genes (*bcrABC*, *emrC*), exhibit low SigB responsiveness, and form biofilm (Maury et al., 2016, 2019; Hafner et al., 2024).

Figure S1

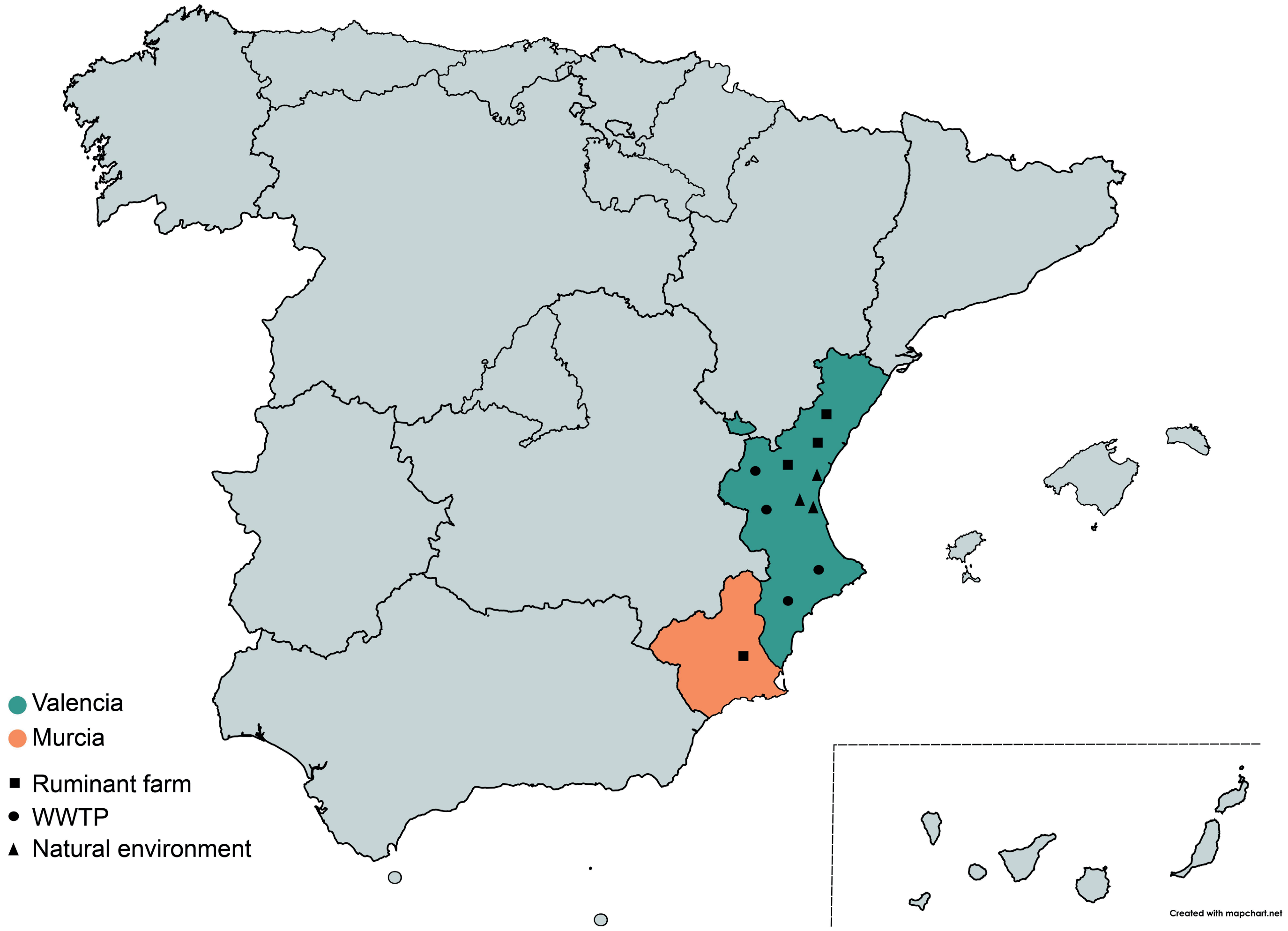
Valencia
Murcia
Ruminant farm
WWTP
Natural environment
Created with mapchart.net

Figure S2

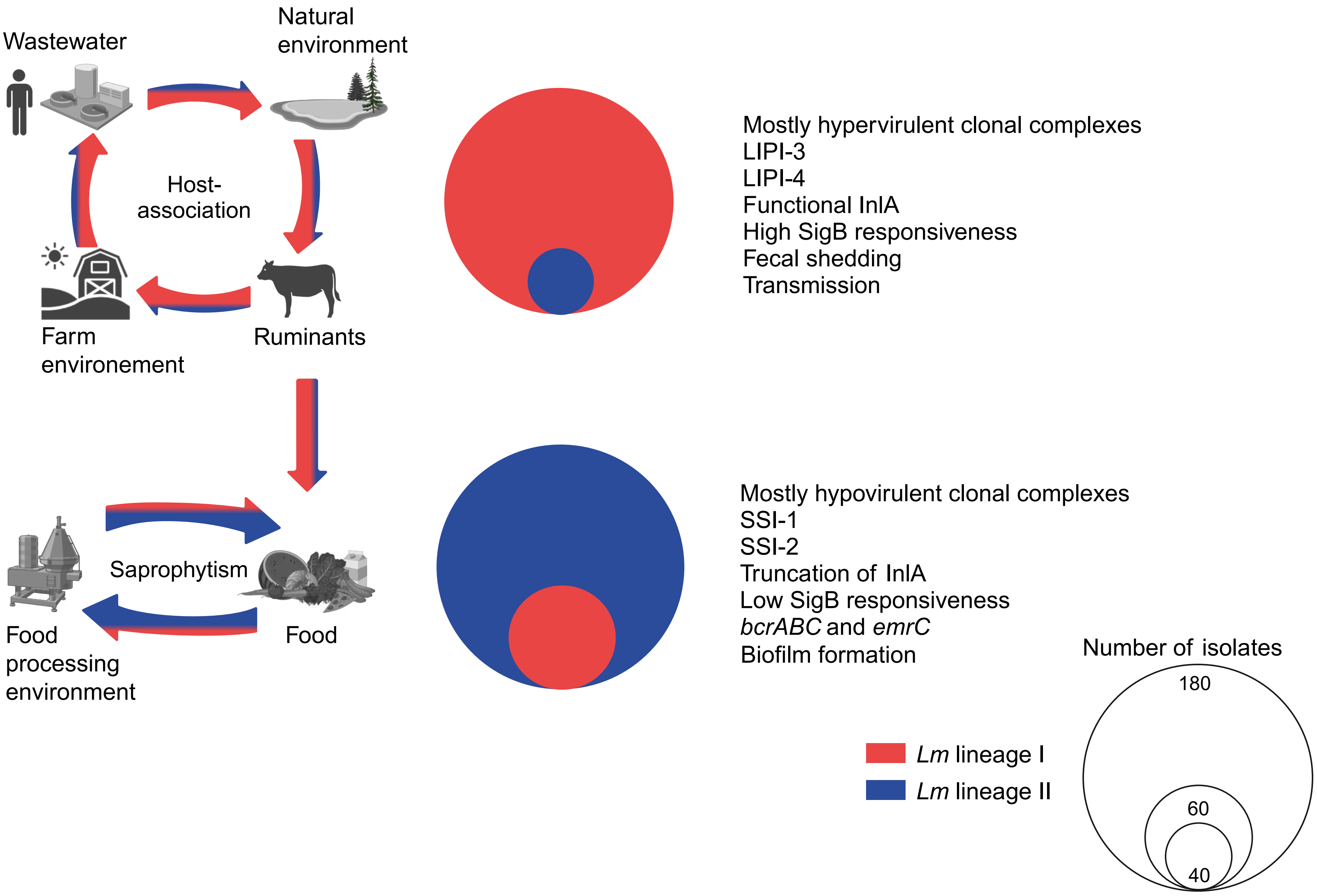

Created in https://BioRender.com
